# Classification of substances by health hazard using deep neural networks and molecular electron densities

**DOI:** 10.1186/s13321-024-00835-y

**Published:** 2024-04-16

**Authors:** Satnam Singh, Gina Zeh, Jessica Freiherr, Thilo Bauer, Isik Türkmen, Andreas T. Grasskamp

**Affiliations:** 1 Department of Sensory Analytics and Technologies, Fraunhofer Institute for Process Engineering and Packaging IVV, Giggenhauser Str. 35, 85354 Freising, Germany; 2 Department of Psychiatry and Psychotherapy, Friedrich-Alexander-Universität Erlangen-Nürnberg, Schwabachanlage 6, 91054 Erlangen, Germany; 3 Computer Chemistry Center, Friedrich-Alexander-Universität Erlangen-Nürnberg, Nägelsbachstr. 25, 91052 Erlangen, Germany

**Keywords:** Electron density, Machine learning, Computational chemistry, Health hazard, 3D-UNet

## Abstract

**Abstract:**

In this paper we present a method that allows leveraging 3D electron density information to train a deep neural network pipeline to segment regions of high, medium and low electronegativity and classify substances as health hazardous or non-hazardous. We show that this can be used for use-cases such as cosmetics and food products. For this purpose, we first generate 3D electron density cubes using semiempirical molecular calculations for a custom European Chemicals Agency (ECHA) subset consisting of substances labelled as hazardous and non-hazardous for cosmetic usage. Together with their 3-class electronegativity maps we train a modified 3D-UNet with electron density cubes to segment reactive sites in molecules and classify substances with an accuracy of 78.1%. We perform the same process on a custom food dataset (CompFood) consisting of hazardous and non-hazardous substances compiled from European Food Safety Authority (EFSA) OpenFoodTox, Food and Drug Administration (FDA) Generally Recognized as Safe (GRAS) and FooDB datasets to achieve a classification accuracy of 64.1%. Our results show that 3D electron densities and particularly masked electron densities, calculated by taking a product of original electron densities and regions of high and low electronegativity can be used to classify molecules for different use-cases and thus serve not only to guide safe-by-design product development but also aid in regulatory decisions.

**Scientific contribution:**

We aim to contribute to the diverse 3D molecular representations used for training machine learning algorithms by showing that a deep learning network can be trained on 3D electron density representation of molecules. This approach has previously not been used to train machine learning models and it allows utilization of the true spatial domain of the molecule for prediction of properties such as their suitability for usage in cosmetics and food products and in future, to other molecular properties. The data and code used for training is accessible at https://github.com/s-singh-ivv/eDen-Substances.

## Introduction

In the field of product development, it is necessary to identify compounds with specific properties as early as possible to minimize non-methodical trial-and-error approaches and consequently reduce development costs. Consumers want new products, such as cosmetics to exhibit desirable, characteristic hedonic properties, e.g., particular odors. At the same time, it is of the highest priority that new products are safe for customers’ health and the environment. The situation is similar for food products and their ingredients, where it is imperative to identify substances that are considered hazardous to health early in the development cycle and avoid their use.

Especially as part of the European Green Deal from the EU Commission [1], the chemical strategy aims to ban chemicals that are harmful to the consumers or the environment. Thus, having a generalized automated system that can help in identifying such substances is key to overcoming this challenge. For this purpose, regulatory bodies such as the European Chemicals Agency (ECHA) and the European Food Safety Authority (EFSA) monitor and maintain a list of substances that can be utilized for various use-cases [2, 3]. This problem can be well defined as a binary classification task that is well suited for an artificial neural network (ANN), not least due to the complex nature of the data.

ANNs have previously used molecular structure relationships to classify substances as carcinogenic [4–6] or to predict molecular properties [7, 8]. In cheminformatics, molecular structures are often represented using specific notations, such as InChI [9] (International Chemical Identifier) or SMARTS [10] (SMILES ARbitrary Target Specification), or, more popularly, with SMILES [11] (Simplified Molecular Input Line Entry Specification) representations, which are a subset of SMARTS. Another method of encoding molecular structures and features is the SELFIES [12] (Self-Referencing Embedded Strings) notation, which is used in various machine learning tasks such as predicting molecular properties or generating new structures, among other applications. These rule-based methods have the benefit of being rather straightforward to generate and easy to understand by chemists. Such representations have therefore been used extensively with machine learning in previous works, like those to generate new molecular structures [13–17]. Additionally, other encoding schemes, such as molecular graphs [15, 18–20], have been widely combined with machine learning to predict molecular properties, like toxicity [21, 22], medical activity in drug discovery [23–26], or even to predict the odor of molecules [27–31].

We assert that such a 2D-representation of molecules is insufficient to model the true spatial domain of the molecule and, as such, at best can roughly approximate properties rooted in the 3D-structure of a molecule. The SMILES notation, for example, has several drawbacks, such as a lack of standard aromaticity handling [32] or no standard method for generating canonical representations with various implementations consisting of implementational variations. On the one hand, this can yield multiple SMILES notations for a single structure [32–34]. On the other hand, there are molecules that cannot easily be defined by graph models, such as those with delocalized bonds, for example, in metal carbonyl complexes [33]. This is also the case for molecules whose atomic arrangements are not fixed in 3D space, making meaningful graph representations difficult to generate. Additionally, while 2D-representations are adequate when calculating charges or polar surface areas for quick classification, gaining a deeper understanding of a molecule’s interaction in binding pockets of receptors necessitates 3D information about its shape, for example in use-cases involving aroma and olfaction.

**Fig. 1:**
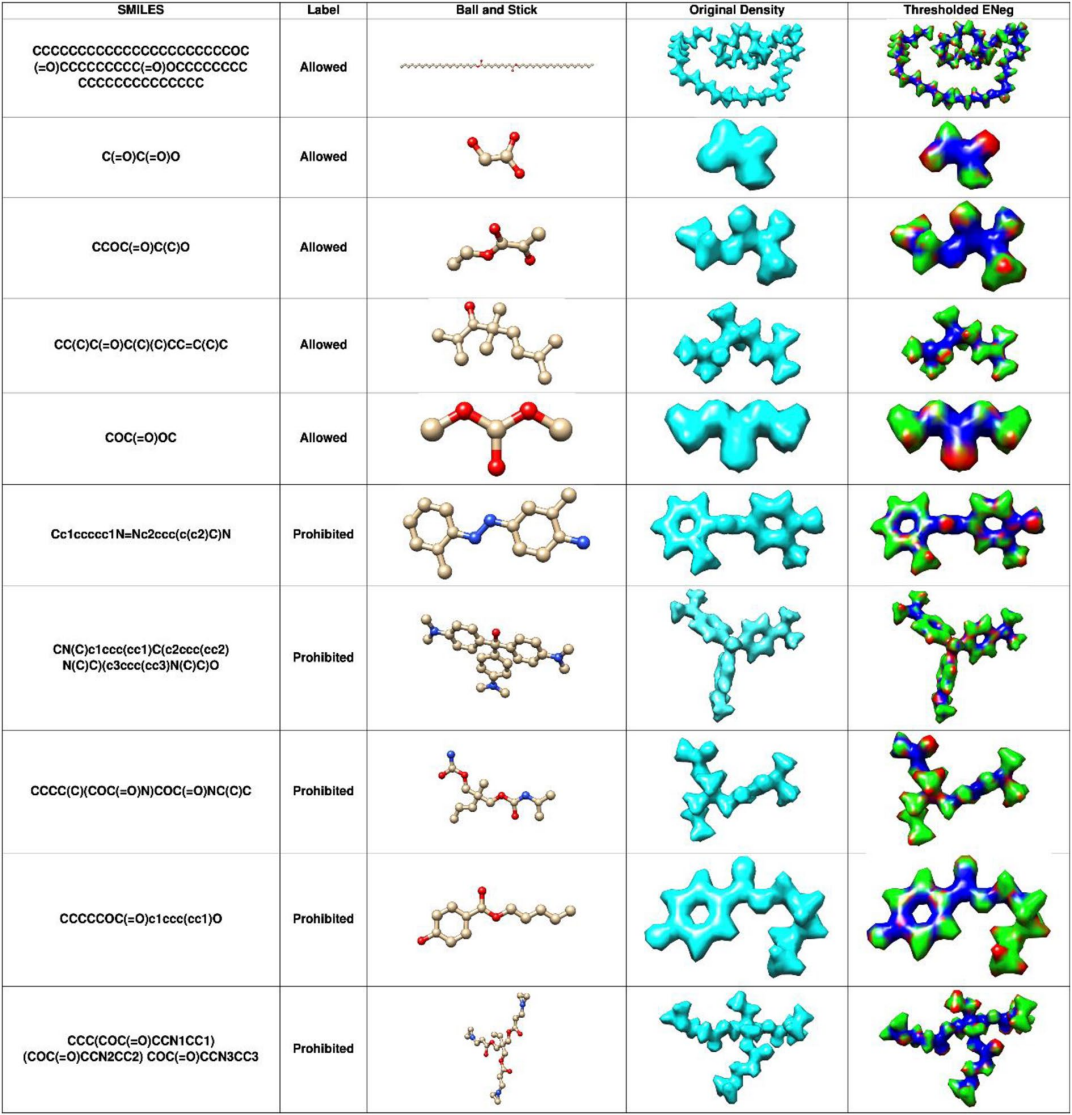
Several molecules sampled from the custom ECHA cosmetics subset for both allowed and prohibited classes and their isomeric SMILES, ball-and-stick, electron density and electronegativity map formats. The electronegativity values have been overlaid on the molecular structure and then divided into three classes based on their percentile threshold values. The blue region shows regions of high electronegativity and hence these voxels are marked as value 2. Red shows regions of low electronegativity and these voxels are marked as 1. All other voxels are marked as 0 and shown as green

This can be seen in Fig. 1, where the SMILES strings do not convey the complex structures of molecules compared to their 3D structures. Recent works have used 3D representation of molecules, like projecting a 3D molecular graph from its 2D structure [35], using 3D molecular conformations [36–38], or the representation of molecules in 3D coordinate space [39–43], and our method takes inspiration from these. In order to overcome the aforementioned limitations, we developed a machine learning pipeline that aims to learn molecular features which are as closely related to the true physics of a molecule as possible without depending on intermediate representations, such as graphs or molecular fingerprints.

Particularly, for understanding toxicity, molecular structure, exposure duration and concentration play crucial roles. Molecules interact with the mammalian body through direct or indirect means, including disruptions in the balance of signal molecules and interactions with receptors such as through shifts in electron densities (chemical reactions) or fitting into receptor pockets leading to various downstream signaling outcomes like tissue degradation and cell mutations [44–46]. Additionally, the mechanism of interaction with surrounding molecules and thereby, toxicity depends on the molecule’s properties. Reactivity of a molecule, for example, determines a molecule’s likelihood to donate or accept electrons and one very common measure to a molecule’s reactivity is its electron density [47, 48]. High electron density sites tend to donate electrons, while low electron density sites are prone to accept electrons. This understanding provides a basis for evaluating a molecule’s potential reactivity and interaction with its surroundings.

For this purpose, we use 3D electron densities as training data for a deep artificial neural network (DNN) pipeline to allow capturing of the spatial features of molecules, which are rooted in quantum physics. We base our method on the core hypothesis of Density Functional Theory (DFT), which postulates that knowing the electron density of a molecule allows direct derivation of various other molecular properties, such as electrostatic potentials, energies, or dipole structures [49–51]. In our pipeline, we use segmented local electronegativity maps of chemical compounds that can be used to identify sites of high and low electronegativity based on a threshold derived from their percentile values. Voxels, i.e., a single unit of 3D grid of size (1 × 1 × 1)—consisting of the electron density or electronegativity values at the location with electronegativity values higher than the 90th percentile were marked as class 2, i.e., high strength electronegative sites, while those less than the 10th percentile were marked as class 1, i.e., low strength electronegative sites, and the remaining voxels were denoted as belonging to class 0, i.e., medium strength electronegative site. These are commonly considered as active sites where reactions would take place [51–55]. Thus, together with electron density distributions they can be used for the classification of compounds into chemical substances that are allowed, i.e., are not hazardous and those that are health hazards and hence prohibited for the two use-cases. Examples of the 3D electron density representation of molecules, and their corresponding ternary electronegativity maps are shown in Fig. 1. Thus, to predict if a substance is hazardous or non-hazardous, our work relies on structural similarity, encompassing both the molecular structure and electron densities, to known hazardous and non-hazardous substances.

## Results and discussion

**Fig. 2:**
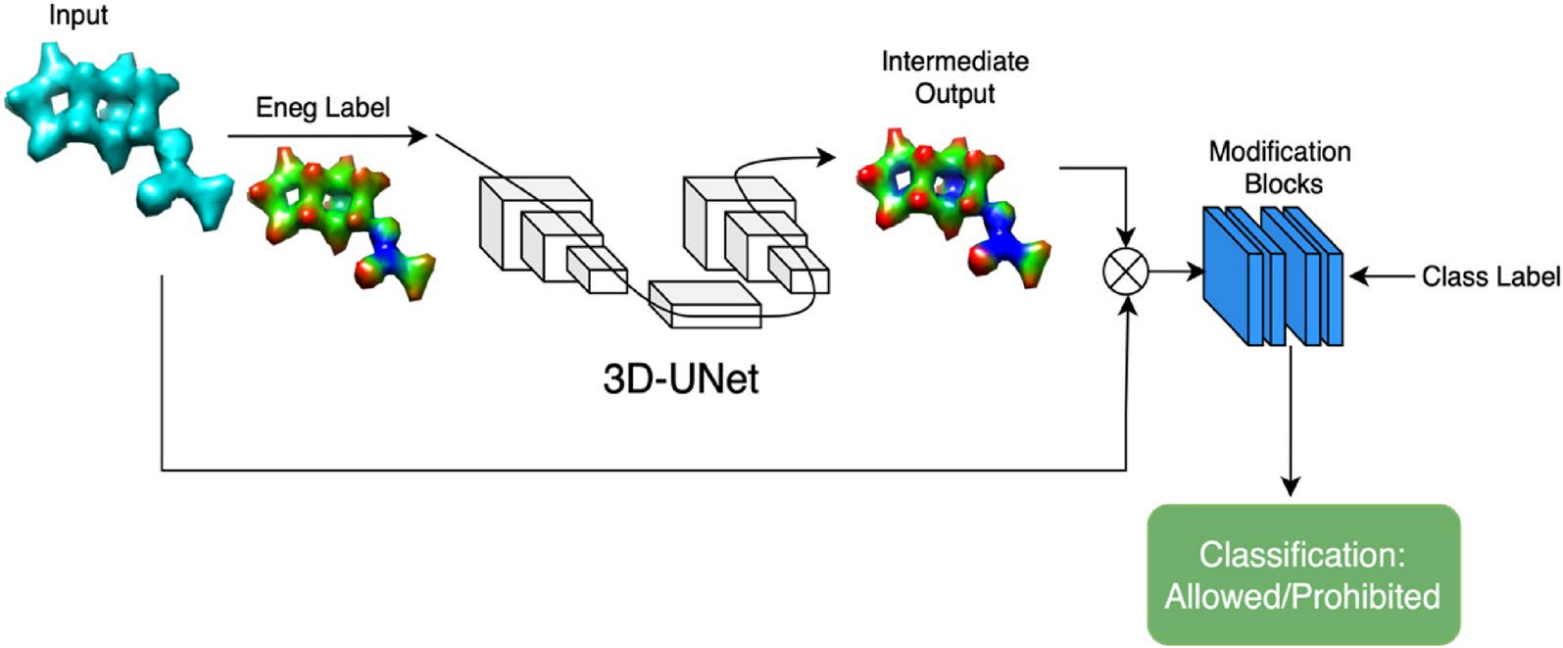
Overview of the electron density pipeline. The modification block consists of 1 × 1 convolution followed by adaptive max pooling, batch normalization and two fully connected layers. The resulting segmentation cube from the UNet is multiplied and passed through the modified block. The fully connected layer assigns class probabilities for the given input using the provided class labels

The pipeline for classifying molecules into two categories is shown in Fig. 2.

The neural network receives information from two sources: a CSV file provides the main class labels (1 for "allowed/non-hazardous" and 0 for "prohibited/hazardous" class) and electronegativity cube files are used as a secondary label to identify specific regions using the 90th upper and 10th lower percentile values denoting high and low reactivity. Electron density cubes and their electronegativity maps are initially fed into the 3D-UNet, producing an intermediate segmentation result, as displayed in Fig. 2. Following this, a 1 × 1 convolution block is applied to reduce channel count and this result is used to mask and highlight specific electron density regions by multiplying the input densities with the intermediate output, followed by batch normalization and adaptive max pooling layers. Finally, two fully connected layers generate probabilities, determining the class of the sample.

### Classification on ECHA dataset

**Fig. 3:**
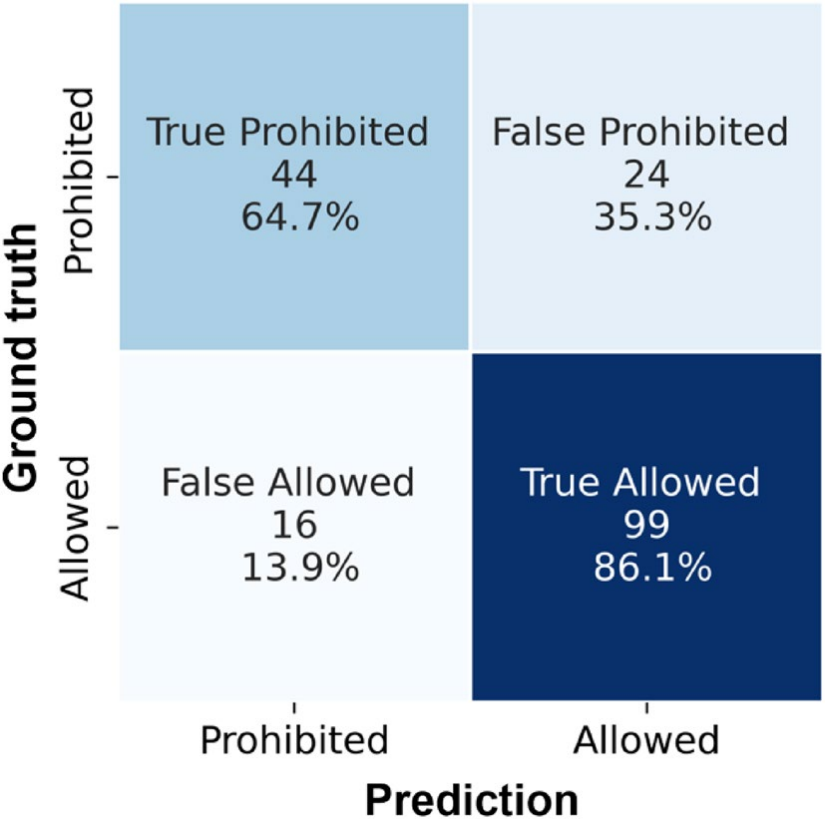
Confusion matrix for classification on the ECHA cosmetics dataset

**Fig. 4:**
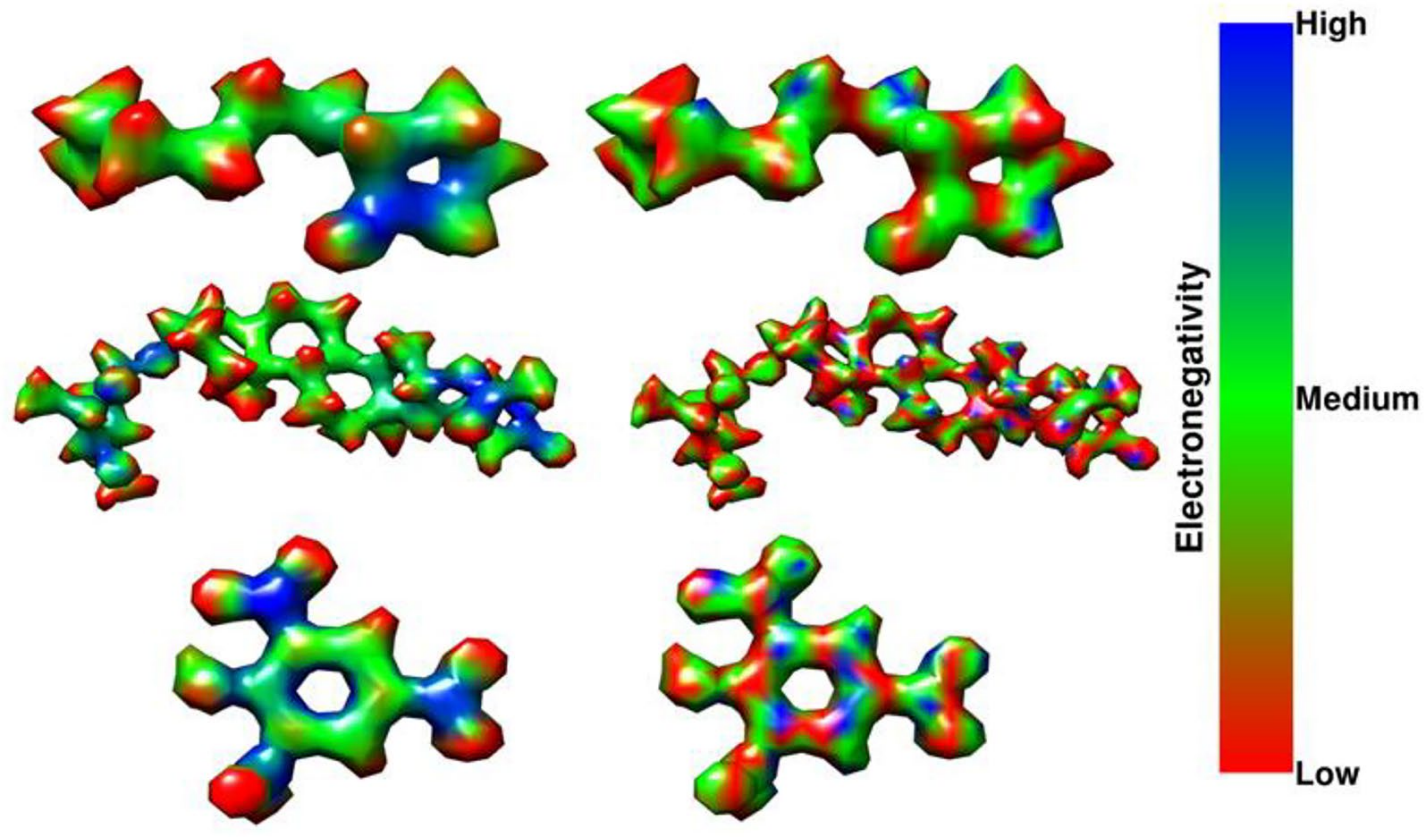
Electronegativity regions for three molecules from the test set along with their corresponding predicted segmentation. The SMILES strings for the compounds are CCCCCCCC1CCCC1 = O, C[C@H]1[C@@H]([C@H](C[C@@H](O1)O[C@H]2CC[C@]3([C@@H](C2)CC[C@@H]4[C@@H]3CC[C@]5([C@@]4(C[C@@H]([C@@H]5C6 = CC(= O)OC6)OC(= O)C)O)C)C)OC)O and c1c(cc(c(c1N(= O) = O)O)N(= O) = O)N(= O) = O respectively

The ECHA dataset consisted of 1356 training samples divided into 855 from the allowed class and 501 belonging to the prohibited/hazardous class. The validation set comprised of 330 samples, where 208 datapoints were from the allowed class and 122 from the prohibited class. Finally, the test set consisted of 183 test samples, divided into 115 samples from the allowed class and 68 from the prohibited class. Table 1 shows the results achieved for classification of molecules into allowed/non-hazardous and prohibited/hazardous classes. Fig. 3 shows the confusion matrix, and examples of the segmented electronegativity regions randomly sampled from the test set are shown in Fig. 4. Furthermore, we performed fivefold cross validation on this dataset to ensure that the performance metrics are not due to a favorable train-test split; these results are shown in Table 2. The averaged dice coefficient values for the model on the test set are shown in Table 3 along with the dice coefficients for CompFood. The low dice coefficient values for classes 1 and 2 are somewhat expected given the fewer number of voxels that are assigned those classes compared to the dominant class. Overall, however, the network seems to be able to handle not only the imbalance in the two classes of allowed and prohibited, but also provides a high classification accuracy of 78.1% for this use-case.

**Table 1:** Results of the classification of molecules into prohibited, i.e., hazardous category (class 0) and allowed, i.e., non-hazardous (class 1) for the ECHA cosmetics test set (higher is better)

Class	Precision	Recall	F1 score	Support
Class 0	0.73	0.65	0.69	68
Class 1	0.80	0.86	0.83	115
Accuracy			0.78	183
Macro Acc.	0.77	0.75	0.76	183
Weighted Acc.	0.78	0.78	0.78	183

Chance prediction would be 62.8% for ECHA dataset

**Table 2:** To ensure that the accuracies achieved for the ECHA dataset were not due to favorable train-test split, we also performed a 5-fold cross validation on the entire dataset. The classification accuracy and weighted F1 scores per fold are summarized here (higher is better)

Fold	Accuracy (%)	Weighted F1 score
0	77.445	0.8136
1	72.826	0.7955
2	73.250	0.7838
3	75.820	0.7982
4	76.366	0.8080
Average	75.141	0.7998

**Table 3:** Average generalized dice scores on the test sets for the ECHA and CompFood datasets (higher is better)

	ECHA dataset	Comp food dataset
Class 0	0.8305	0.8473
Class 1	0.1960	0.1802
Class 2	0.2537	0.3991

### Classification on CompFood dataset

The results of classification on the CompFood dataset are shown in Table 4. CompFood dataset consisted of 4262 train samples, divided into 2271 allowed and 1991 prohibited datapoints. Moreover, the validation set consisted of 474 samples, divided into 239 allowed and 235 prohibited substances. Finally, the test set consisted of a total of 836 substances, divided into 463 allowed and 373 prohibited substances.

**Table 4:** Classifications of molecules into prohibited, i.e., hazardous category (class 0) and allowed, i.e., non-hazardous (class 1) for the CompFood test set (higher is better)

Class	Precision	Recall	F1 score	Support
Class 0	0.58	0.72	0.64	373
Class 1	0.72	0.58	0.64	463
Accuracy			0.64	836
Macro Acc.	0.65	0.65	0.64	836
Weighted Acc.	0.66	0.64	0.64	836

Chance prediction would be 55.4% for this case

**Fig. 5:**
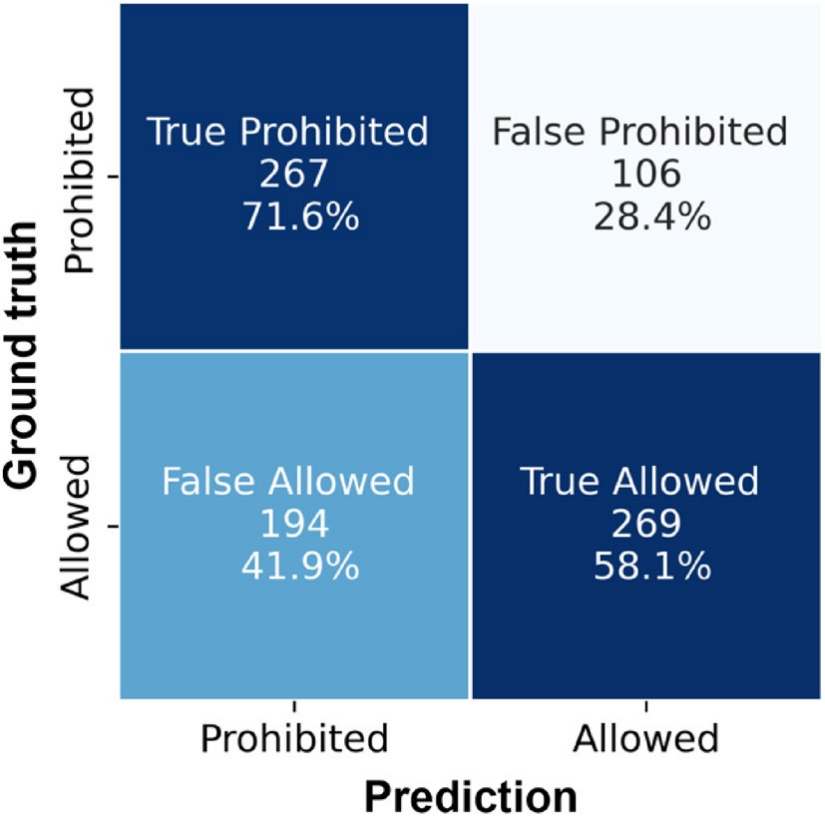
Confusion matrix for classification on the CompFood dataset

**Fig. 6:**
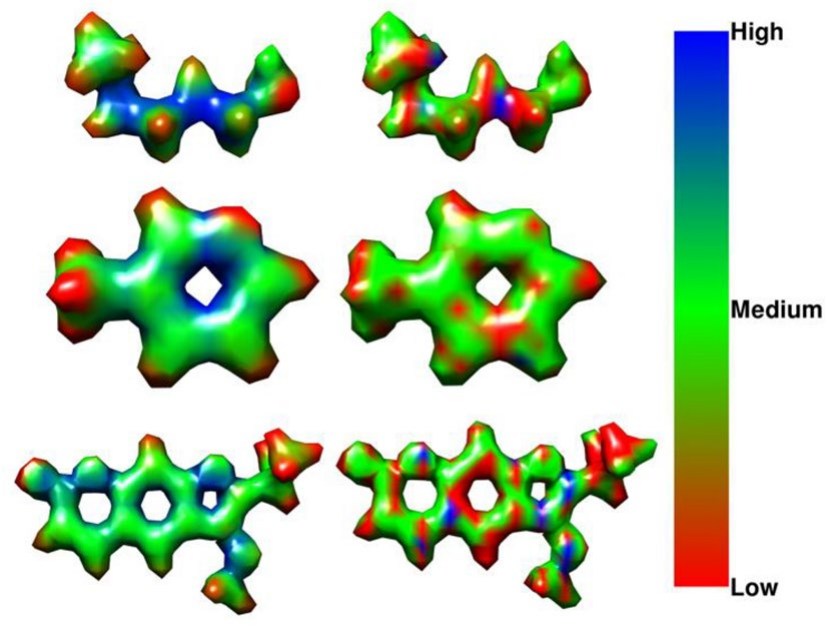
Electronegativity regions for three molecules from the test set for the CompFood dataset along with their corresponding predicted segmentation using the modified 3D-UNet pipeline. The SMILES strings for the three molecules are CCCCCCS, Cc1cccnc1 and COc1c(oc2cc3OC(=O)C=Cc3cc12)C(C)C respectively

The confusion matrix for the results is shown in Fig. 5. The classification report indicates that while the overall classification accuracy of 64.11% is much higher than chance, there is still scope for significant improvement of the model. Examples from the segmentation of electronegativity files sampled randomly from the test set molecules are shown in Fig. 6 and the average dice coefficient values across the test set are shown in Table 3. Like the previous case, here, the dice coefficient for the two under-represented classes (class 1–2) is less than that of the majority class (class 0), which is somewhat expected.

Overall, we show that our model is able to achieve up to 78.1% binary class accuracy for the ECHA dataset and 64.1% accuracy for the CompFood dataset. Using thresholded electronegativity maps as reactive sites of the molecules and thus as weights for the electron densities allows specific spatial regions within the molecule to be highlighted, which would not be possible with a 2D representation. This enables the network to use only these electron densities for making a decision on the molecules being in the hazardous/non-hazardous class.

We hypothesize that the difference in performance between the two datasets could be attributed to the presence of more ‘complex’ compounds in the CompFood dataset, which might pose challenges for the network to learn. Molecular complexity, however, is a complex topic which is not in the current scope of work, but we calculate the fraction of chiral centers [56] (FCC) to gain insight into the two datasets. The CompFood dataset consists of compounds with higher FCC values (1.61 Mean, 3.86 SD) than the ECHA dataset (0.87 Mean, 2.21 SD), and thus this could be one reason for the difference in performance. Moreover, another possible scenario could be the effect of concentration that is not currently considered in our approach. For example, Ethanol is one compound in the CompFood dataset, that is labelled as prohibited due to it being considered carcinogenic but present in wide range of cosmetics and thus allowed in ECHA dataset. Two strategies for our future work to counter this would be to introduce a weight/penalty for misclassifying ‘complex’ compounds in the loss function along with considering concentration of the substances below which they would be considered belonging to the ‘allowed’ class.

Our prototype pipeline thus allows the molecular properties to be established directly based on the physics of the molecule without depending on intermediate steps, such as lossy fingerprint translation. This approach opens up various other future possibilities, such as molecular structure replacement by identifying sites that contribute to the reactive nature of the molecule and testing if the replacement structure leads to a change in the hazardous/non-hazardous class assignment. Among others, our future work will focus on improving the performance of the network and transferring this to other properties, such as the logP (octanol–water partition coefficient) of the underlying molecule that can be verified in a laboratory setting.

## Conclusions

We demonstrate a machine learning pipeline that uses 3D electron density and electronegativity information to segment regions of high, medium, and low electronegativity and classify substances as health hazardous or non-hazardous with considerably higher than chance accuracy. For this purpose, we first created a custom dataset of cube files by performing semi-empirical molecular calculations for all molecules present in the ECHA dataset consisting of molecules that are considered health hazardous and hence prohibited or non-hazardous and thus allowed for cosmetic use. These cube files were used to train a modified 3D-UNet to segment 3-class electronegativity maps that were derived by setting an upper and lower threshold on the electronegativities before being used for classification of the given molecules.

Moreover, we show that this kind of approach can be used for various use-cases, for example, in cosmetics or food products by performing the same data generation, pre-processing, and training steps on the CompFood dataset consisting of substances considered carcinogenic or safe in a binary class problem that were compiled from the OpenFoodTox, GRAS and FooDB datasets. With our work, we aim to demonstrate that a prototype pipeline that uses electron densities and deep neural networks can be used in the product development cycle as an early predictor to reduce future trial and errors, as well as aid in regulatory decisions.

## Methods

### Data generation

Initially, a list of substances prohibited for use in cosmetic products under EU Cosmetic Products Regulation was retrieved from the European Chemicals Agency’s database for Information on Chemicals [2]. The prohibited substances are those chemicals that are classified as carcinogenic, mutagenic, or toxic for reproduction by the European Union and hence considered a health hazard. Additionally, a second list of allowed substances was created that do not belong to this list, i.e., those not restricted by ECHA. For this purpose, we sampled a disjoint set of molecules with molecular weight < 400 Da from the ZINC [57] database. In this work, we use ‘hazardous’ and ‘prohibited’ interchangeably and similarly, ‘non-hazardous’ and ‘allowed’ are used interchangeably.

For creating the training dataset, in a first step CAS numbers were used to query PubChem via their REST API [58] to retrieve the isomeric SMILES representations of the substances in our prohibited and allowed categories. Using RDKit [59], these SMILES strings were converted to 3D structures, optimized using the Merck Molecular Force Field (MMFF) [60] and exported to mol2 files, from which we generated input files for the EMPIRE [61] software. We used EMPIRE and the AM1S [62] Hamiltonian to perform geometry optimizations and to generate an electronic wave-function for each molecule. The wave-function was then used to generate electron density, electronegativity and electron-affinity cube files using the eh5cube software from Cepos [61]. The final dataset consisted of 3D-electronegativity and the 3D electron-affinity cube files for each of the 1869 molecules, of which 1178 are allowed and 691 are prohibited, and this was then divided randomly into stratified train, validation, and test sets in the approximate ratio of 70:20:10.

The labels for classifying molecules into “allowed” or “prohibited” classes were one-hot encoded with allowed = 1 and prohibited = 0. To map physical properties onto the feature space, a local property map of electronegativity was used as a secondary label, as follows. Since high (local) electronegativity is correlated to high (local) reactivity [52], we derive a ternary reactivity mask from the electronegativity cube files for regions of high, medium and low local reactivity. To categorize reactivity, voxels with electronegativity values above the 90th percentile were labeled as class 2, signifying high reactive sites. Conversely, those below the 10th percentile were labeled as class 1 for low reactive sites. All remaining voxels were designated as class 0, indicating medium reactivity. Examples of the 3D electron density representation of molecules, and their corresponding ternary electronegativity maps, are shown in Fig. 1.

For the generation of the compiled food dataset, three independent datasets were combined. Firstly, the OpenFoodTox [3] from EFSA containing 4201 substances with their CAS numbers was downloaded. These consisted of 3409 substances found in food products labeled as ‘Positive’ denoting a carcinogenic compound, 375 as ‘No Data’, i.e., either no carcinogenicity assessment was made or no studies are available, 209 labelled as ‘Negative’, 51 as ‘Not Determined’, i.e., no clear conclusion could be made, 32 as ‘Other’, 37 as ‘Ambiguous’ and 88 as ‘Not applicable’. Thus, from this dataset, 3409 substances were selected for the prohibited class. To assign substances as allowed, only those belonging to the Negative class were chosen, i.e., 209 substances were assigned as allowed. To balance out the class distribution, additional substances were added to the allowed class from the GRAS database [63] that provided 381 compounds that are generally recognized as safe for consumption, such as in the form of a food additive and a further 3167 “non-hazardous” substances were randomly sampled from the FooDB dataset [64] that consists of a comprehensive collection of food compounds and their associated chemical compositions, nutrients and flavors. Using our data preparation steps we generated a total of 5572 cube files, with 2599 compounds belonging to the prohibited class and 2973 to the allowed class. This data was then subdivided into train-test-validation sets in the ratio of 75:15:10 with the aim of training an ANN pipeline for this binary classification problem using 3D electron density and electronegativity representation of these substances.

### Loss functions, evaluation and hyperparameter search

For classification of molecules into allowed/prohibited classes, a sum of cross entropy (CE) loss between ground-truth and predicted class labels along with the dice loss between the original and predicted electronegativity maps was used, denoted as $${L}_{ovr}={L}_{ce}+ {L}_{gen\_dice}$$. Since the thresholded electronegativity voxels lead to a very imbalanced distribution of classes, we used generalized dice loss instead of the simple dice loss [65]. This allows introducing a weighting scheme for the different classes that are underrepresented. For our implementation, we used the generalized dice coefficient implementation from the Monai library [66]. This loss function is defined as $${L}_{gen\_dice}= 1-(1/(y^2+ \varepsilon))*((2*y* y \,\hat + \varepsilon)/(y+ y \,\hat + \varepsilon))$$. The CE loss used for training is defined as $${L}_{ce}=- \sum_{i=1}^{2}{w}_{i}{y}_{i}{\text{log}}\left(\widehat{{y}_{i}}\right)$$. Here, $$y$$ corresponds to the ground truth labels and $$\widehat{{y}_{i}}$$ corresponds to the predicted labels. $${w}_{i}$$ are the weights for class $$i$$ shown in Supp. Table 3. Moreover, to account for class imbalance, especially classification on the ECHA dataset, the CE loss was provided with class weights for the ECHA dataset that were optimized along with the other hyperparameters. For the CompFood dataset, however, the CE class weights were found to be almost the same, which would make sense since the classes are sufficiently balanced for the classification task. The performance of the models was determined by calculating the accuracy on the test set, along with their confusion matrices. The models were trained using Pytorch [67] library (version 2.0.0) for Python using a cluster of 4 Nvidia Quadro 8000 GPUs. The hyper-parameters for both trainings were selected by performing hyperparameter search using Optuna [68] and a Tree-structured Parzen Estimator. The final parameters are listed in Table 5.

**Table 5:** Hyperparameters chosen for training of the neural networks are shown here

Hyper parameter	ECHA dataset	CompFood dataset
EPOCHS	38	14
Learning rate	0.000844	0.0002409
Batch size	20	12
Rate decay	0.1 every 35 epochs	–
Weight decay	2.57e−7	0.000186
Feature size	28	4
Filter size	4	4
Weight class 0	0.65127	1.24926
Weight class 1	1.34672	1.1977
Final layer neurons	16	32

## Data Availability

The data and code used for training is accessible at https://github.com/s-singh-ivv/eDen-Substances/. The algorithm as well as all other further preprocessing steps are described in detail in the Method section.
